# Circulating soluble LOX-1 and patient prognosis after an acute coronary syndrome

**DOI:** 10.1136/heartjnl-2025-326315

**Published:** 2025-09-16

**Authors:** Alexandru Schiopu, Sara Svedlund, Gayathri Narasimhan, Bi Juin Loong, Troels Yndigegn, Vijayalakshmi Varma, Emily L Ongstad, Isabel Goncalves, Anna Collén, Jan Nilsson, Li-Ming Gan

**Affiliations:** 1 Department of Translational Medicine, Lund University, Malmö, Sweden; 2 Institute of Cellular Biology and Pathology Nicolae Simionescu, Bucharest, Romania; 3 Department of Molecular and Clinical Medicine, University of Gothenburg, Gothenburg, Sweden; 4 Ribocure Pharmaceuticals AB, Gothenburg, Sweden; 5 Department of Clinical Sciences Malmö, Lund University, Malmö, Sweden; 6 Department of Cardiology, Skåne University Hospital, Lund, Sweden; 7 Department of Clinical Sciences Lund, Lund University, Lund, Sweden; 8 Translational Science and Experimental Medicine, Research and Early Development, Cardiovascular, Renal and Metabolism, BioPharmaceuticals R&D, AstraZeneca, Gaithersburg, Maryland, USA; 9 Bioscience Cardiovascular, Research and Early Development, Cardiovascular, Renal and Metabolism, BioPharmaceuticals R&D, AstraZeneca, Gaithersburg, Maryland, USA; 10 Department of Cardiology, Skåne University Hospital, Malmö, Sweden; 11 Projects, Research and Early Development, Cardiovascular, Renal and Metabolism, BioPharmaceuticals R&D, AstraZeneca, Gothenburg, Sweden

**Keywords:** Acute Coronary Syndrome, Inflammation, Heart failure, Cohort Studies, Biomarkers

## Abstract

**Background:**

The lectin-like oxidised low-density lipoprotein receptor-1 (LOX-1) mediates atherosclerotic plaque inflammation and vulnerability. On activation, LOX-1 sheds its extracellular domain into the circulation as soluble LOX-1 (sLOX-1). sLOX-1 is markedly elevated in patients with acute coronary syndrome (ACS).

**Methods:**

We prospectively assessed the associations between plasma sLOX-1 and the development of heart failure (HF), major adverse cardiovascular events (MACE) and coronary and left ventricular (LV) dysfunction in two cohorts of patients with ACS. The first cohort comprised 524 patients recruited during the acute index event at the coronary care unit of Skåne University Hospital, Malmö, Sweden. The second cohort included 363 patients with ACS treated with acute percutaneous intervention at Sahlgrenska University Hospital, Gothenburg, Sweden. Additionally, we examined the anti-inflammatory effects of LOX-1 blockade in vitro using human umbilical vein endothelial cells (HUVECs).

**Results:**

In the first cohort, acute-phase sLOX-1 was associated with incident HF and MACE independently of cardiovascular risk factors, revascularisation and medication (HR per 1-SD sLOX-1 increase: 1.57 (95% CI: 1.10 to 2.23; p=0.012) for HF and 1.36 (1.08 to 1.71; p=0.009) for MACE). Elevated sLOX-1 was also associated with lower LV ejection fraction and accelerated remodelling, as measured by echocardiography at 1-year post-ACS. In the second cohort, sLOX-1 was negatively associated with left anterior descending coronary artery flow reserve and LV systolic function, and positively correlated with soluble markers of systemic inflammation and cardiac overload at 4 and 16 weeks post-ACS. In vitro, antibody-mediated LOX-1 blockade prevented oxidised low-density lipoprotein-induced HUVEC activation.

**Conclusions:**

Elevated plasma sLOX-1 at baseline and during follow-up is associated with incident HF and MACE, as well as cardiac and coronary dysfunction in patients with ACS. As plasma sLOX-1 levels may reflect the intensity of LOX-1 expression on vascular and immune cells, these findings support LOX-1 as a potentially important therapeutic target to improve prognosis in patients with ACS.

**WHAT IS ALREADY KNOWN ON THIS TOPIC**

- The oxidised low-density lipoprotein receptor (LOX)-1 is upregulated in inflammatory states and contributes to the development and destabilisation of atherosclerotic plaques.
- Circulating soluble LOX-1 (sLOX-1) levels are elevated in acute coronary syndrome (ACS) compared with stable coronary artery disease.

**WHAT THIS STUDY ADDS**

- In a cohort of 524 patients with ACS, elevated acute-phase sLOX-1 was prospectively associated with increased long-term risk of left ventricular dysfunction, heart failure and major adverse cardiovascular events.
- In an independent cohort of 363 ACS survivors, high sLOX-1 at 4 and 16 weeks post-ACS was linked to reduced coronary flow reserve and impaired left ventricular function.

**HOW THIS STUDY MIGHT AFFECT RESEARCH, PRACTICE OR POLICY**

- These findings support sLOX-1 as a prognostic biomarker and promote LOX-1 as a potential therapeutic target, strengthening the rationale for ongoing clinical trials of LOX-1–blocking monoclonal antibodies in patients with ACS.

## Introduction

 Lectin-like oxidised low-density lipoprotein receptor-1 (LOX-1) is the main receptor for oxidised low-density lipoprotein (oxLDL) on endothelial cells.^1^ Binding of oxLDL to LOX-1 initiates oxLDL uptake and degradation,^1^ but also leads to endothelial cell activation, dysfunction and apoptosis.^2^ LOX-1 is also present on inflammatory-activated smooth muscle cells, platelets and macrophages.^3^ Previous clinical and experimental studies have indicated a major role for LOX-1 in atherogenesis and atherosclerotic plaque destabilisation, leading to acute coronary syndrome (ACS).^3^

Cell-bound LOX-1 is upregulated by oxLDL, inflammatory cytokines such as TNFα, angiotensin II, shear stress and advanced glycation end-products^2 4^ and is elevated under proatherogenic conditions such as diabetes, hypertension, dyslipidaemia and autoimmune disease.^3–5^ Activation of LOX-1 leads to intracellular signalling pathways that accelerate atherogenesis through foam cell formation, vascular smooth muscle cell proliferation, platelet activation, reduced nitric oxide bioavailability, decreased cyclic GMP levels, generation of reactive oxygen species and collagen degradation.^4 5^ LOX-1 is present in atherosclerotic lesions, and previous studies have suggested a role for the receptor in intraplaque neovascularisation, plaque growth and rupture.^3 6^ LOX-1 was also found to be upregulated in the heart after experimental ischaemia/reperfusion^7^ and was associated with markers of inflammation, oxidative stress and cardiomyocyte apoptosis.^8–10^ Administration of an anti-LOX-1 antibody prior to coronary occlusion in rats reduced cardiac inflammation and infarction size and improved cardiac recovery.^8^

oxLDL and inflammatory cytokines stimulate LOX-1 upregulation on cell surface and shedding of the extracellular domain through proteolytic cleavage of its NECK domain.^11^ LOX-1 shedding has been found to be stimulated by interleukin (IL)-18 through a mechanism involving ADAM10 (a disintegrin and metalloproteinase domain-containing protein 10),^12^ and plasma levels of soluble LOX-1 (sLOX-1) are thought to reflect the abundance of the receptor on cell surface.^11^ sLOX-1 is highly elevated in patients with ACS compared with healthy individuals and patients with stable coronary artery disease and has been proposed as a possible early diagnostic biomarker, alongside cardiac troponin.^13 14^ Small prospective studies have previously suggested possible associations between high plasma sLOX-1 and major adverse coronary events in patients with ACS.^15 16^ In the largest sLOX-1 study to date, Kraler *et al* recently found an independent association between plasma sLOX-1 and mortality in a large cohort of 2639 patients with ACS.^17^

Despite experimental studies suggesting a causative role of LOX-1 in the exacerbation of post-ischaemic left ventricular (LV) dysfunction, no clinical studies have explored the potential link between sLOX-1 and post-ACS heart failure (HF). We combined prospective and cross-sectional cohort studies to examine whether high plasma levels of sLOX-1 in the acute phase of an ACS and during follow-up are associated with LV dysfunction, remodelling, cardiac overload and incident HF. We also assessed whether plasma sLOX-1 is related to coronary artery microvascular dysfunction, systemic inflammation and incident MACE post-ACS.

## Methods

Detailed material and methods description can be found in the online supplemental material.

### Study populations and outcomes

The study included two separate cohorts of patients with ACS. The first cohort (cohort 1) has been described previously^18^ and includes 524 consecutively enrolled patients with available baseline plasma samples, collected within 24 hours of admission with a confirmed ACS diagnosis (online supplemental figure 1). The patients have been recruited at the coronary care unit of Skåne University Hospital Malmö, Sweden between October 2008 and December 2012. ACS was defined as unstable angina or acute MI, according to the universal myocardial infarction (MI) definition.^19^ Cohort 1 was followed prospectively for a median (IQR) period of 2.20 (1.15–3.27) years for incident HF and 2.08 (1.07–3.14) years for incident MACE. Participants were followed from baseline until the first occurrence of the outcome of interest, death or the end of the follow-up period (31 December 2012), whichever came first. Participants who did not experience an event were censored at the time of death or at the end of follow-up. Time-to-event analyses were performed using Cox proportional hazards models, accounting for censoring. MACE was defined as a composite of hospitalisation with an ACS diagnosis or cardiovascular (CV) death. Data from the Swedish Hospital Discharge Register and the Swedish Cause of Death Register were used to identify events. MI was defined by International Classification of Diseases (ICD) 10 codes I21 and I22, unstable angina by code I20 and HF by code I50. CV death was defined as death due to MI, ischaemic heart disease, cardiac arrest, atrial fibrillation, HF and ischaemic or haemorrhagic stroke, defined by the ICD10 codes I20-25, I46, I48, I50 and I60-69.

The second cohort (cohort 2) consisted of 363 patients with ACS who have undergone acute percutaneous coronary intervention (PCI) at index hospitalisation at Sahlgrenska University Hospital, Gothenburg, Sweden between February 2010 and October 2013. The patients underwent follow-up examinations of cardiac function and coronary flow reserve (CFR) by echocardiography within 4 and 16 weeks of PCI and donated plasma samples at both follow-up visits (online supplemental figure 2). Patients who were unable to comply with the study protocol were excluded.

### sLOX-1 analysis

sLOX-1 was measured within 24 hours from admission in cohort 1, and at the 4 weeks and 16 weeks follow-up visits in cohort 2. For both cohorts, the analysis of sLOX-1 in plasma was performed at the Science for Life Laboratory, Uppsala, Sweden, by the Proximity Extension Assay technique, as previously described.^18 20^ Data for sLOX-1 are presented as arbitrary units (au).

## Results

### Baseline characteristics

The differences in clinical and biomarker baseline characteristics between the outcome groups in cohort 1 are presented in table 1. The patients who were hospitalised with a HF diagnosis during follow-up were older and had a higher prevalence of hypertension, diabetes and previous ACS or HF than those without. These patients also had lower estimated glomerular filtration rate and were prescribed statins to a lower extent at discharge. There were no significant differences in the type of index coronary event or in baseline troponin T (TnT) levels (table 1). A similar pattern was observed in patients that suffered MACE during follow-up versus those who did not (table 1), as previously published in this cohort.^18^ A lower proportion of these patients underwent revascularisation and were prescribed ACE inhibitor/angiotensin receptor blockers, beta-blockers and statins at discharge (table 1).

**Table 1:** Differences in clinical characteristics at baseline between patients with ACS in cohort 1, divided by outcome

Characteristics	All patients	Incident HF	No incident HF	P value	Incident MACE	No incident MACE	P value
	N=524	n=41	n=483		n=87	n=437	
Age, years, median (IQR)	67 (59–77)	77 (72–85)	66 (58–76)	<0.001	77 (66–85)	66 (58–74)	<0.001
Male gender, n (%)	383 (73.1)	28 (68.3)	355 (73.5)	0.470	61 (70.1)	322 (73.7)	0.493
Hypertension, n (%)	285 (54.4)	34 (82.9)	251 (52.0)	< 0.001	56 (64.4)	229 (52.4)	0.041
Smoking, n (%)	132 (25.2)	10 (24.4)	122 (25.3)	0.902	18 (20.7)	114 (26.1)	0.290
Diabetes, n (%)	126 (24.0)	20 (48.8)	106 (21.9)	< 0.001	24 (27.6)	102 (23.3)	0.397
BMI, kg/m^2^, median (IQR)	27 (24–30)	27 (24–30)	27 (24–30)	0.614	26 (24–30)	27 (24–30)	0.196
eGFR, mL/min/1.73m^2^, median (IQR)	72 (53–95)	46 (32–64)	74 (56–96)	<0.001	55 (34–76)	75 (57–97)	<0.001
Index cardiac event				0.378			0.124
STEMI	179 (34.2)	11 (26.8)	168 (34.8)	0.295	27 (31.0)	152 (34.8)	0.501
NSTEMI	295 (56.3)	24 (58.5)	271 (56.1)	0.753	56 (64.4)	239 (54.7)	0.097
UA	50 (9.5)	6 (14.6)	44 (9.1)	0.246	4 (4.6)	46 (10.5)	0.086
Previous cardiac event, n (%)							
HF	54 (10.3)	15 (36.6)	39 (8.1)	<0.001	18 (20.7)	36 (8.2)	<0.001
ACS	151 (28.8)	19 (46.3)	132 (27.3)	0.010	39 (44.8)	112 (25.6)	<0.001
Coronary angiography, n (%)				0.020			0.043
Left main stenosis	40 (7.6)	5 (12.2)	35 (7.2)		12 (13.8)	28 (6.4)	
1-vessel no left main	133 (25.4)	3 (7.3)	130 (26.9)		16 (18.4)	117 (26.8)	
2-vessel no left main	93 (17.7)	9 (22.0)	84 (17.4)		14 (16.1)	79 (18.1)	
3-vessel no left main	112 (21.4)	13 (31.7)	99 (20.5)		17 (19.5)	95 (21.7)	
Non-significant stenosis	40 (7.6)	1 (2.4)	39 (8.1)		3 (3.4)	37 (8.5)	
Revascularisation, n (%)	362 (69.1)	23 (56.0)	339 (70.1)	0.061	46 (52.8)	316 (72.3)	<0.001
PCI	309 (59.0)	19 (46.3)	290 (60.0)	0.087	43 (49.4)	266 (60.9)	0.048
CABG	53 (10.1)	4 (9.7)	49 (10.1)	0.937	3 (3.4)	50 (11.4)	0.020
Discharge medication, n (%)							
ACEi/ARB	463 (88.4)	35 (85.4)	428 (88.6)	0.508	68 (78.2)	395 (90.4)	0.003
Beta blockers	482 (92.0)	39 (95.1)	443 (91.7)	0.462	72 (82.8)	410 (93.8)	0.001
Statins	504 (96.2)	35 (85.4)	469 (97.1)	< 0.001	74 (85.1)	430 (98.4)	<0.001
ASA	504 (96.2)	39 (95.1)	465 (96.3)	0.657	81 (93.1)	423 (96.8)	0.237
P2Y12 antagonists	443 (84.5)	32 (78.0)	411 (85.1)	0.218	77 (88.5)	366 (83.8)	0.173
TnT, ng/L, median (IQR)	366 (63–1291)	496 (94–1248)	358 (55–1303)	0.453	379 (96–1290)	364 (55–1297)	0.337
sLOX-1, au, median (IQR)	5.0 (4.7–5.4)	5.28 (5.1–5.8)	4.99 (4.6–5.4)	<0.001	5.2 (4.9–5.6)	5.0 (4.6–5.4)	<0.001

ACEi, ACE inhibitors; ACS, acute coronary syndrome; ARB, angiotensin receptor blockers; ASA, acetylsalicylic acid; au, arbitrary units; BMI, body mass index; CABG, coronary artery bypass graft; eGFR, estimated glomerular filtration rate; HF, heart failure; MACE, major adverse cardiovascular events; NSTEMI, non-ST-elevation myocardial infarction; PCI, percutaneous coronary intervention; sLOX-1, lectin-like oxidised low-density lipoprotein receptor-1; STEMI, ST-elevation myocardial infarction; TnT, troponin T; UA, unstable angina.

Baseline sLOX-1 was significantly higher in patients hospitalised for HF or MACE compared with those that remained event-free during follow-up (table 1). Among CV risk factors at baseline, the presence of smoking was a strong independent determinant of elevated sLOX-1 levels in a mutually adjusted multivariable linear regression analysis (online supplemental table 1). In bivariate Spearman’s correlation analyses, sLOX-1 was not correlated with age (r=0.060; p=0.172) or TnT (r=0.047; p=0.286) and presented only a weak inverse correlation with kidney function (r=–0.090; p=0.039).

Baseline characteristics for cohort 2 are presented in online supplemental table 2. Patients in this cohort were younger (median (IQR) 63 (25–82) vs 67 (59–77)) and included a higher percentage of males (82.8% vs 73.1%) compared with cohort 1. Patients in cohort 2 also had a lower prevalence of hypertension (42.9% vs 54.4%) and diabetes (12.2% vs 24.0%) compared with cohort 1, but the percentage of smokers (25.2% vs 25.2%) and median (IQR) BMI (27 (19–41) vs 27 (24–30)) were identical in the two cohorts.

### High baseline sLOX-1 at index ACS is associated with increased risk for incident HF and MACE during follow-up

**Figure 1:**
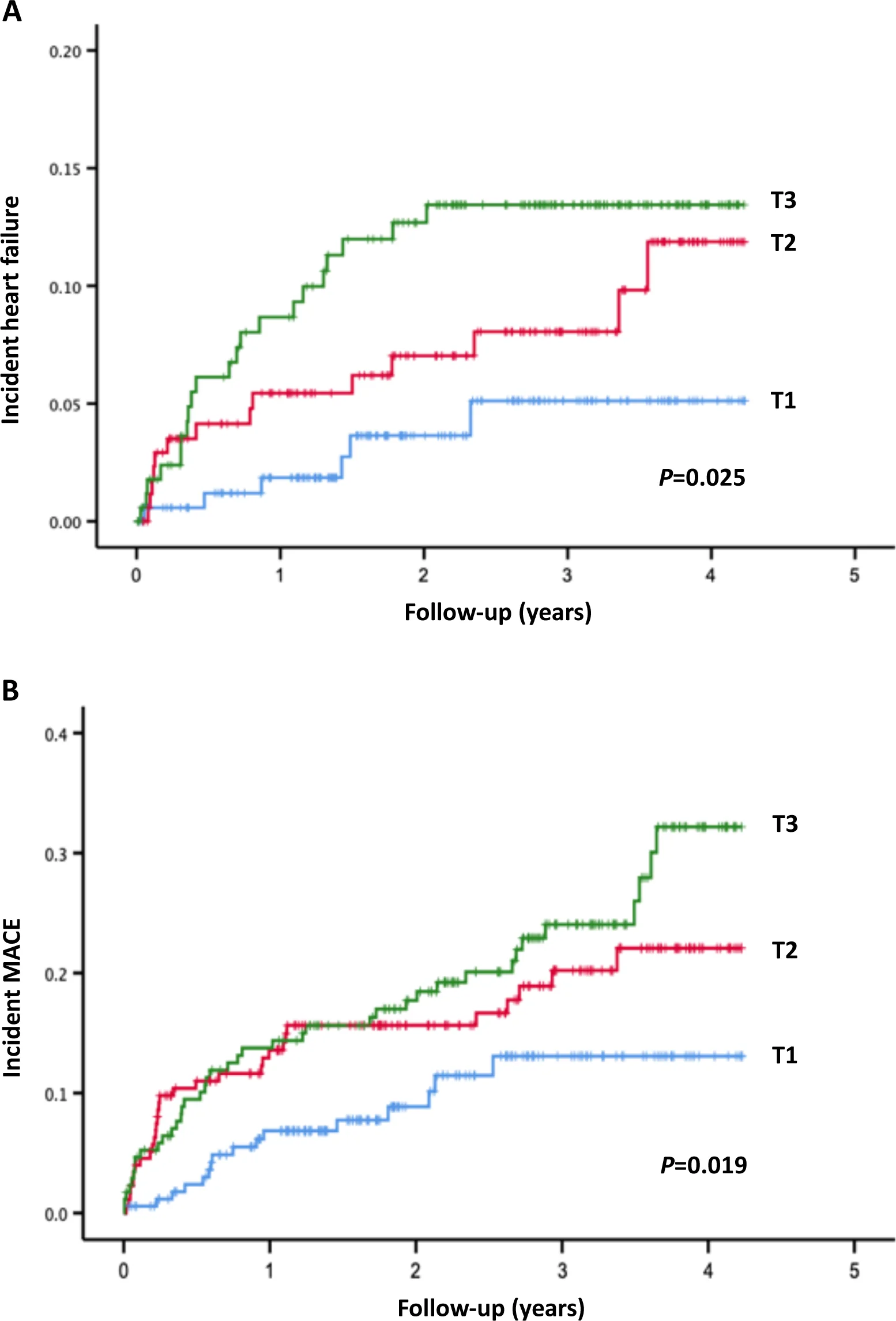
Acute-phase sLOX-1 predicts incident MACE and HF during follow-up in patients with ACS. Kaplan-Meier curves of incident HF (**A**) and MACE (**B**) during follow-up by baseline sLOX-1 tertiles. The p values refer to the difference in incident events across tertiles (unadjusted log-rank test). ACS, acute coronary syndrome; HF, heart failure; MACE, major adverse cardiovascular events; sLOX-1, soluble lectin-like oxidised low-density lipoprotein receptor-1; T, tertile.

In Kaplan-Meier survival analyses with log-rank tests, cohort 1 patients with plasma sLOX-1 in the highest tertile (T3) had a significantly higher incidence of HF (figure 1A) and MACE (figure 1B) during follow-up, compared with patients in the lowest tertile (T1). We recorded a steep increase in incident HF cases during the first 2 years post-ACS among patients within the highest sLOX-1 tertile, but there was no difference between the top two tertiles regarding incident MACE during this period (figure 1A,B). In a multivariable Cox proportional hazard analysis adjusted for age, sex, hypertension, diabetes, smoking, kidney function, prevalent HF and ACS, sLOX-1 was independently associated with incident HF during follow-up, with a 54% increased risk per 1-SD increase in baseline plasma sLOX-1 concentration (HR 1.54 (95% CI: 1.09 to 2.17; p=0.015), table 2, Model 2). Further adjustment for revascularisation and statin treatment at discharge, parameters that also differed significantly between the outcome groups at baseline (table 1), did not affect the relationship (table 2, Model 3). We found similar associations between plasma sLOX-1 and incident MACE, independent of all considered potential confounders (table 2, Models 1–3), with HR 1.36 (95% CI: 1.08 to 1.71; p=0.009) for 1-SD baseline sLOX-1 increase in the fully adjusted model.

**Table 2:** Linear associations between baseline sLOX-1, incident HF and MACE during follow-up in cohort 1

Event	Model	HR (95% CI)*	P value
HF (n=41)	1	1.55 (1.14 to 2.10)	0.005
	2	1.54 (1.09 to 2.17)	0.015
	3	1.57 (1.10 to 2.23)	0.012
MACE (n=87)	1	1.34 (1.09 to 1.67)	0.007
	2	1.28 (1.03 to 1.59)	0.029
	3	1.36 (1.08 to 1.71)	0.009

Cox proportional hazards regression analysis, using the following adjustment models: Models: (1) Age and sex. (2) Age, sex, hypertension, smoking, diabetes, kidney function (eGFR), previous ACS and previous HF. (3) Model 2 with the addition of revascularisation and statins at discharge. Incident MACE was additionally adjusted for ACEi/ARB and beta blockers at discharge.

* HR expressed per 1-SD LOX-1 increase. The sLOX-1 values were log-transformed and standardised as Z-scores prior to analysis.

ACEi, ACE inhibitors; ACS, acute coronary syndrome; ARB, angiotensin receptor blockers; eGFR, estimated glomerular filtration rate; HF, heart failure; LOX-1, lectin-like oxidised low-density lipoprotein receptor-1; MACE, major adverse cardiovascular events; sLOX-1, soluble lectin-like oxidised low-density lipoprotein receptor-1.

### High baseline sLOX-1 is associated with LV systolic dysfunction and remodelling at 1-year post-ACS

To further confirm the relationship between sLOX-1 and incident HF, we investigated the correlations between sLOX-1 at inclusion and echocardiographic parameters of cardiac structure and function at inclusion and at 1 year after the index event. At baseline, we found a positive correlation between plasma sLOX-1 and body surface area (BSA)-indexed left ventricular end-systolic volume, but no significant correlation with left ventricular ejection fraction (LVEF) and BSA-indexed left ventricular end-diastolic volume (table 3). Baseline plasma sLOX-1 did not differ between patients with ACS with and without prevalent HF (Mann-Whitney U Test, p=0.257), suggesting that pre-existing HF did not influence sLOX-1 levels. In contrast, high baseline sLOX-1 was associated with cardiac remodelling and deterioration of LV function post-ACS, characterised by significantly lower LVEF and larger LV volumes at the 1-year follow-up (table 3). We found no relationships between baseline sLOX-1 and parameters of LV hypertrophy and diastolic dysfunction (table 3).

**Table 3:** Correlations between baseline sLOX-1, cardiac structure and function in cohort 1

	Index ACS		1-year follow-up	
	r*	P value*	r*	P value*
LV systolic function				
LVEF	–0.217	0.063	–0.263	0.009
LV volumes				
LVEDV	0.176	0.134	0.204	0.045
LVEDVi	0.191	0.103	0.240	0.020
LVESV	0.243	0.037	0.244	0.016
LVESVi	0.251	0.031	0.271	0.008
LV diastolic function				
LAVi	–0.047	0.638	0.103	0.295
LVMi	0.097	0.332	–0.019	0.849
MVE	0.077	0.481	0.040	0.694
MVDT	0.030	0.790	–0.061	0.546
e’	–0.137	0.150	–0.149	0.135
E/e’ > 15		0.154†		0.668†

Associations between sLOX-1 measured at inclusion and echocardiographic parameters of cardiac structure and function during index hospitalisation (n=74) and at the 1-year follow-up (n=97).

* Spearman’s correlation analysis between continuous echocardiographic variables and sLOX-1 levels.

† Mann-Whitney U Test comparing sLOX-1 levels between patients with E/e’ >15 (indicating diastolic dysfunction) and patients with E/e’ <15.

ACS, acute coronary syndrome; BSA, body surface area; e’, early mitral annulus tissue Doppler velocity; E/e’, ratio E through e’; LAVi, BSA-indexed left atrial volume; LV, left ventricular; LVEDV, left ventricular end-diastolic volume; LVEDVi, BSA-indexed LVEDV; LVEF, left ventricle ejection fraction; LVESV, left ventricular end-systolic volume; LVESVi, BSA-indexed LVESV; LVMi, BSA-indexed left ventricular mass; MVDT, mitral valve early Doppler deceleration time; MVE, mitral valve early Doppler velocity; r, correlation coefficient; sLOX-1, soluble lectin-like oxidised low-density lipoprotein receptor-1.

### Elevated sLOX-1 during follow-up is associated with impaired LAD CFR, LV systolic dysfunction and increased systemic inflammation

In cohort 2**,** males had significantly lower sLOX-1 levels at the 4-week follow-up in a mutually adjusted multivariable linear regression analysis (online supplemental table 3). At the first follow-up visit within 4 weeks post-ACS, elevated sLOX-1 was associated with coronary artery microvascular dysfunction and with LV dysfunction, witnessed by significant negative correlations with left anterior descending (LAD) CFR, LVEF and stroke volume at rest and during adenosine stress (table 4). Additionally, sLOX-1 was positively correlated with plasma levels of IL-6, IL-16, IL-18 and myeloperoxidase, suggestive of elevated systemic inflammatory activity. Plasma sLOX-1 was also positively correlated with matrix metalloproteinases (MMP)1, MMP7 and MMP12, critical regulators of extracellular matrix turnover and inflammatory signalling and with plasma N-terminal prohormone of brain natriuretic peptide (NT-proBNP), biomarker of cardiac overload with negative prognostic value. The correlations between sLOX-1 and LAD CFR, LVEF and inflammatory biomarkers remained significant at the 16-week visit (table 4), confirming a long-lasting stable relationship between elevated sLOX-1, cardiac and coronary dysfunction during follow-up.

**Table 4:** Correlations between baseline sLOX-1, cardiac function and biomarkers at 4 weeks and 16 weeks post-ACS in cohort 2

	4 weeks		16 weeks	
	r*	P value*	r*	P value *
LV systolic function				
LVEF	–0.139	0.017	–0.137	0.024
CFR in LAD				
CFR	–0.137	0.011	–0.119	0.041
LV volumes				
SV rest	–0.137	0.011	–0.053	0.382
SV hyp	–0.224	0.001	–0.172	0.014
LVEDV	–0.092	0.111	–0.040	0.505
LVESV	–0.028	0.662	–0.010	0.873
LVEDV hyp	–0.144	0.027	–0.039	0.574
LVESV hyp	–0.028	0.662	0.001	0.986
Biomarkers				
IL-6	0.394	0.001	0.321	0.001
IL-16	0.448	0.001	0.419	0.001
IL-18	0.316	0.001	0.329	0.001
MMP-1	0.286	0.001	0.254	0.001
MMP-7	0.195	0.001	0.392	0.001
MMP-12	0.276	0.001	0.363	0.001
MPO	0.539	0.001	0.543	0.001
NT-pBNP	0.235	0.001	0.259	0.001

* Spearman’s correlation analysis between plasma sLOX-1 concentrations and the other analysed variables at the respective time point.

ACS, acute coronary syndrome; CFR, coronary flow reserve; hyp, hyperaemia measurement during adenosine infusion; IL, interleukin; LAD, left anterior descending; LV, left ventricular; LVEDV, left ventricular end-diastolic volume; LVESV, left ventricular end-systolic volume; MMP, matrix metalloprotein; MPO, myeloperoxidase; NT-pBNP, N-terminal prohormone of brain natriuretic peptide; rest, baseline measurement at rest before infusion of adenosine; sLOX-1, soluble lectin-like oxidised low-density lipoprotein receptor-1; SV, stroke volume.

### LOX-1 mediates endothelial cell activation and expression of adhesion molecules

**Figure 2:**
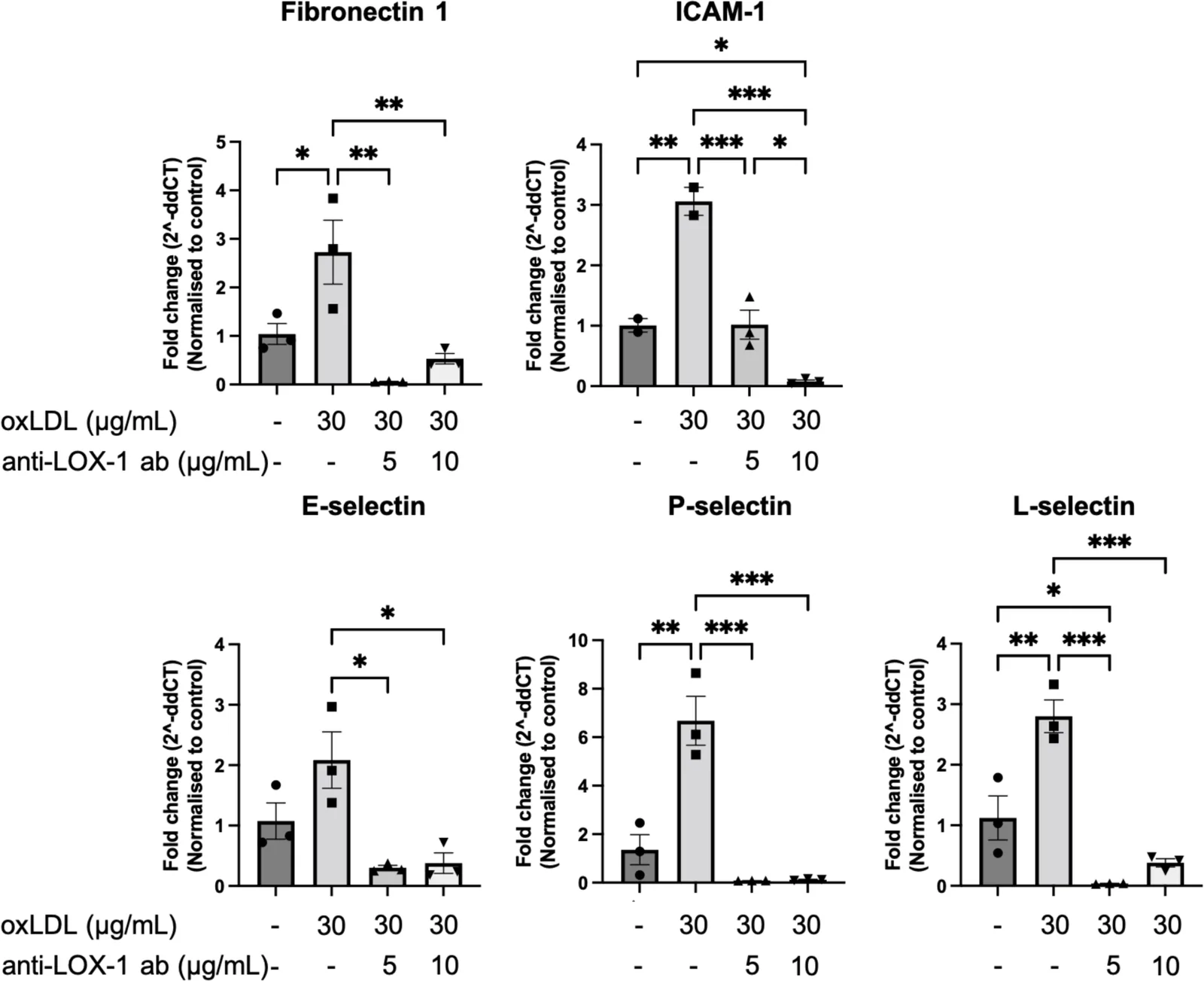
LOX-1 blockade prevents oxLDL-induced endothelial cell activation in vitro. Fibronectin 1, ICAM-1, E-selectin, P-selectin and L-selectin gene expression in HUVECs treated with 30 µg/mL oxLDL, with or without 5 µg/mL or 10 µg/mL anti-LOX-1 antibodies. Gene expression levels are presented in comparison with untreated cells. *p<0.05, **p<0.01, ***p<0.001. ab, antibody; ddCT, delta delta cycle threshold; HUVECs, human umbilical vein endothelial cells; LOX-1, lectin-like oxidised low-density lipoprotein receptor-1; oxLDL, oxidised low-density lipoprotein.

To investigate the potential causal link between elevated sLOX-1 and coronary artery microvascular dysfunction, we treated human umbilical vein endothelial cell (HUVEC) cultures in vitro with 30 µg/mL oxLDL in the presence or absence of 5 µg/mL or 10 µg/mL of an anti-LOX-1 blocking antibody. oxLDL upregulated gene expression of the endothelial cell activation markers fibronectin 1, ICAM-1, P-selectin, L-selectin and E-selectin (figure 2). These effects were reversed by both concentrations of the anti-LOX-1 antibody, supporting a direct role of the receptor in oxLDL-induced endothelial cell activation, expression of leucocyte adhesion molecules and vascular inflammation.

## Discussion

Our study investigates the long-term associations between plasma sLOX-1 and the development of LV dysfunction, remodelling and HF in patients with ACS. We also examined the relationship between sLOX-1, coronary microvascular dysfunction and incident MACE. Prospectively, we demonstrate that patients with elevated acute-phase sLOX-1 exhibit impaired LV function and increased remodelling at 1-year post-ACS, along with a higher long-term risk of developing HF and MACE. In an independent cross-sectional cohort, persistently elevated plasma sLOX-1 during follow-up was associated with coronary and cardiac dysfunction, systemic inflammation and cardiac overload. In vitro, we show that LOX-1 blockade prevents oxLDL-induced activation of human endothelial cells, suggesting that LOX-1–mediated pathways may contribute to coronary microvascular dysfunction in patients with sustained sLOX-1 elevation after ACS.

The link between elevated sLOX-1 and the development of post-ACS HF is supported by previous experimental findings in animal models of myocardial ischaemia and HF. LOX-1 is highly upregulated in rodent and swine hearts following coronary ischaemia or ischaemia/reperfusion. Genetic deletion, antibody-mediated or siRNA-mediated LOX-1 inhibition potently reduced myocardial oxidative stress, apoptosis, inflammation, hypertrophy, fibrosis, infarction size, remodelling and loss of function.^7–9 21 22^ Importantly, LOX-1 overexpression was found to mediate a direct proapoptotic effect of oxLDL on cardiomyocytes.^10^ The deleterious effects of LOX-1-mediated pathways have been further confirmed in animal models of hypertension, where LOX-1 acts synergistically with the angiotensin receptor 1 to induce cardiac remodelling, fibrosis and dysfunction.^23^ LOX-1 was also found to be upregulated in failing rat hearts, and sLOX-1 is elevated in patients with LV dysfunction and hypertrophy.^24^ These studies provide a strong body of evidence supporting the role of LOX-1 as a treatment target to prevent or ameliorate post-ischaemic HF.

sLOX-1 has previously been proposed as a diagnostic biomarker in ACS, as plasma sLOX-1 increases earlier than cardiac troponin and is highly elevated in patients with ACS compared with patients with stable coronary artery disease.^14 17 25^ sLOX-1 was also found to be significantly higher in patients with ACS with complex lesions and with plaque rupture or erosion verified by optical coherence tomography.^25 26^ The prognostic value of sLOX-1 in the context of ACS has previously been investigated in small studies examining the ability of acute-phase sLOX-1 to predict recurrent coronary events and mortality. In a study on 94 patients undergoing PCI for ACS, the 13 patients that subsequently suffered recurrent ACS or death had significantly higher plasma sLOX-1 levels at baseline, and sLOX-1 was a better risk predictor compared with high-sensitivity C-reactive protein (hsCRP) and TnT.^15^ Further, in a cohort of 153 patients with ST-elevation MI, subjects with plasma sLOX-1 at admission above median had increased risk for incident MACE and mortality.^16^ The link between elevated baseline sLOX-1 and mortality has recently been confirmed in the large multicentre Special Program University Medicine-ACS (SPUM-ACS) study, showing that patients with ACS with baseline sLOX-1 in the highest tertile had an approximately twice higher risk for total and CV mortality compared with the lowest tertile. The study also revealed that increasing sLOX-1 from baseline to the 1-year follow-up was directly associated with accelerated coronary plaque progression.^17^ Here, we demonstrate an independent association between acute-phase sLOX-1 and cardiac remodelling, LV dysfunction and incident HF. Importantly, we show in the independent cross-sectional arm that the negative association between plasma sLOX-1 and LVEF is maintained long-term.

Clinical studies evaluating the efficacy of LOX-1 blockade in patients with ACS are already ongoing. MEDI6570 is a high-affinity human IgG1 monoclonal antibody developed to specifically block the binding of various ligands to LOX-1. In a phase 1 placebo-controlled study in 88 patients with type 2 diabetes mellitus (NCT03654313), MEDI6570 was found to be safe and well tolerated and reduced mean plasma sLOX-1 levels by up to 83% after 10 weeks treatment in the multiple ascending doses groups.^27^ Moreover, three doses of MEDI6570 induced a non-significant regression of non-calcified coronary plaque volume compared with placebo (−13.45 mm^3^ vs −8.25 mm^3^), as assessed by CT angiography. The efficiency of MEDI6570 is being investigated in the global phase 2b study GOLDILOX-TIMI 69 (mNCT04610892), a placebo-controlled dose-dependent study in patients with a recent MI. The trial evaluates the ability of LOX-1 blockade with MEDI6570 to reduce non-calcified coronary plaque volume in the most diseased segment and to lower surrogate biomarkers of inflammation. The results of our study and previous experimental data support potential protective effects of LOX-1 blockade against the development of post-ACS cardiac dysfunction. The GOLDILOX-TIMI 69 trial will also investigate the effects of the treatment on the change in LVEF, global longitudinal strain and NT-proBNP during a 9-month follow-up period.

Our results are based on two well-characterised independent cohorts of patients with ACS, permitting evaluation of sLOX-1 levels both within 24 hours of ACS and during follow-up. The strengths of the study include the lack of exclusion criteria for enrolment, the medium-sized patient cohorts and the reproducibility of the associations between sLOX-1 and LV dysfunction in both cohorts. The study also has some notable limitations. Plasma sLOX-1 levels were measured by the Proximity Extension Assay technique and expressed in au. We are therefore not able to make direct comparisons with sLOX-1 levels measured by ELISA in other studies or to determine clinically useful thresholds for risk stratification. It should nevertheless be noted that this limitation extends to other studies measuring sLOX-1 in clinical cohorts, and to date, no clinically useful threshold has been defined. Second, follow-up echocardiographic data at 1 year after the acute event was only available in a preselected subgroup of 97 patients aged ≥75 years from cohort 1, and therefore these data should be interpreted with caution. However, the findings in this subgroup are supported by the association between sLOX-1 and incident HF in the entire cohort 1, as well as by the negative correlations between sLOX-1 and LV function at 4 and 16 weeks in cohort 2. Finally, clinical follow-up data is not available for patients in cohort 2, and we were therefore not able to determine whether the associations between sLOX-1, coronary dysfunction and systemic inflammation also lead to an increased risk of adverse events.

In conclusion, we demonstrate that elevated sLOX-1 levels during both the acute phase and follow-up are associated with LV remodelling and dysfunction, and that acute-phase sLOX-1 independently predicts long-term HF. We also identify significant associations between plasma sLOX-1 and coronary microvascular dysfunction, systemic inflammation and recurrent coronary events (Graphical Abstract). These findings support the potential of sLOX-1 as a prognostic biomarker for residual CV risk in patients with ACS. Alongside previous experimental studies targeting LOX-1–mediated pathways in atherosclerosis and MI, our results offer important clinical rationale for pursuing LOX-1–targeted therapies in this setting. The ongoing Phase 2b trial evaluating the humanised LOX-1-blocking antibody MEDI6570 will provide key insight into the therapeutic potential of LOX-1 inhibition to prevent plaque progression, myocardial dysfunction and to reduce systemic inflammation and cardiac stress in patients with ACS. If successful, the trial could pave the way for future strategies aimed at improving post-ACS outcomes through LOX-1 inhibition.

## Supplementary material

10.1136/heartjnl-2025-326315 online supplemental file 1

10.1136/heartjnl-2025-326315 online supplemental file 2

10.1136/heartjnl-2025-326315 online supplemental figure 1

10.1136/heartjnl-2025-326315 online supplemental figure 2

10.1136/heartjnl-2025-326315 online supplemental table 1

## Data Availability

Data are available upon reasonable request.
