# Supplementary material for: Circulating soluble LOX-1 and patient prognosis after an acute coronary syndrome
Source: Heart. 2025 Sep 16;112(18):e326315. doi: 10.1136/heartjnl-2025-326315 (PMC13539867; doi:10.1136/heartjnl-2025-326315)

Supplemental figure 1. Patient inclusion, exclusion and follow-up echocardiography in cohort 1

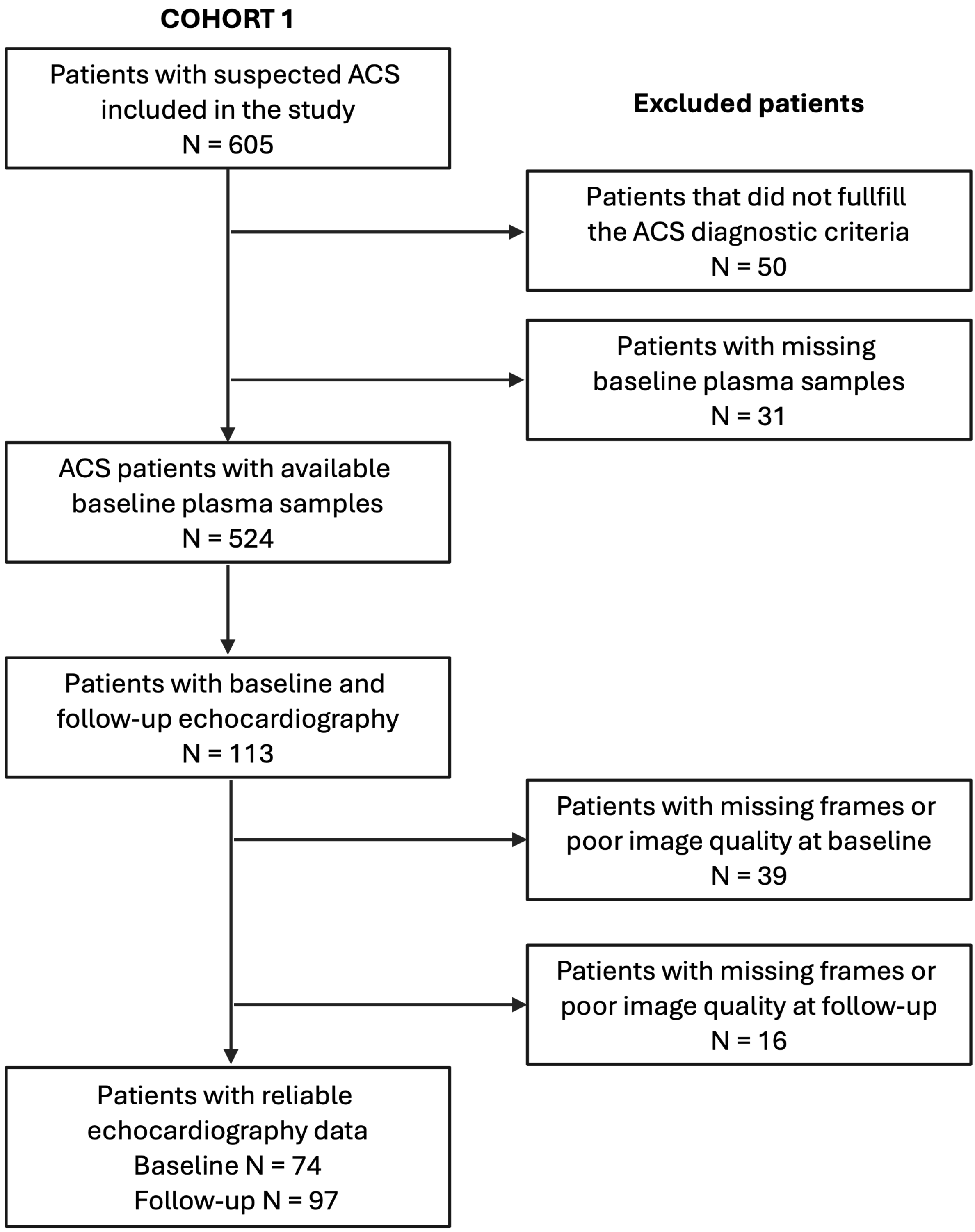

Supplement: Supplementary file 1 [file heartjnl-112-18-s003.pdf]
