# Supplementary material for: Circulating soluble LOX-1 and patient prognosis after an acute coronary syndrome
Source: Heart. 2025 Sep 16;112(18):e326315. doi: 10.1136/heartjnl-2025-326315 (PMC13539867; doi:10.1136/heartjnl-2025-326315)

Supplemental figure 2. ACS patient follow-up in cohort 2

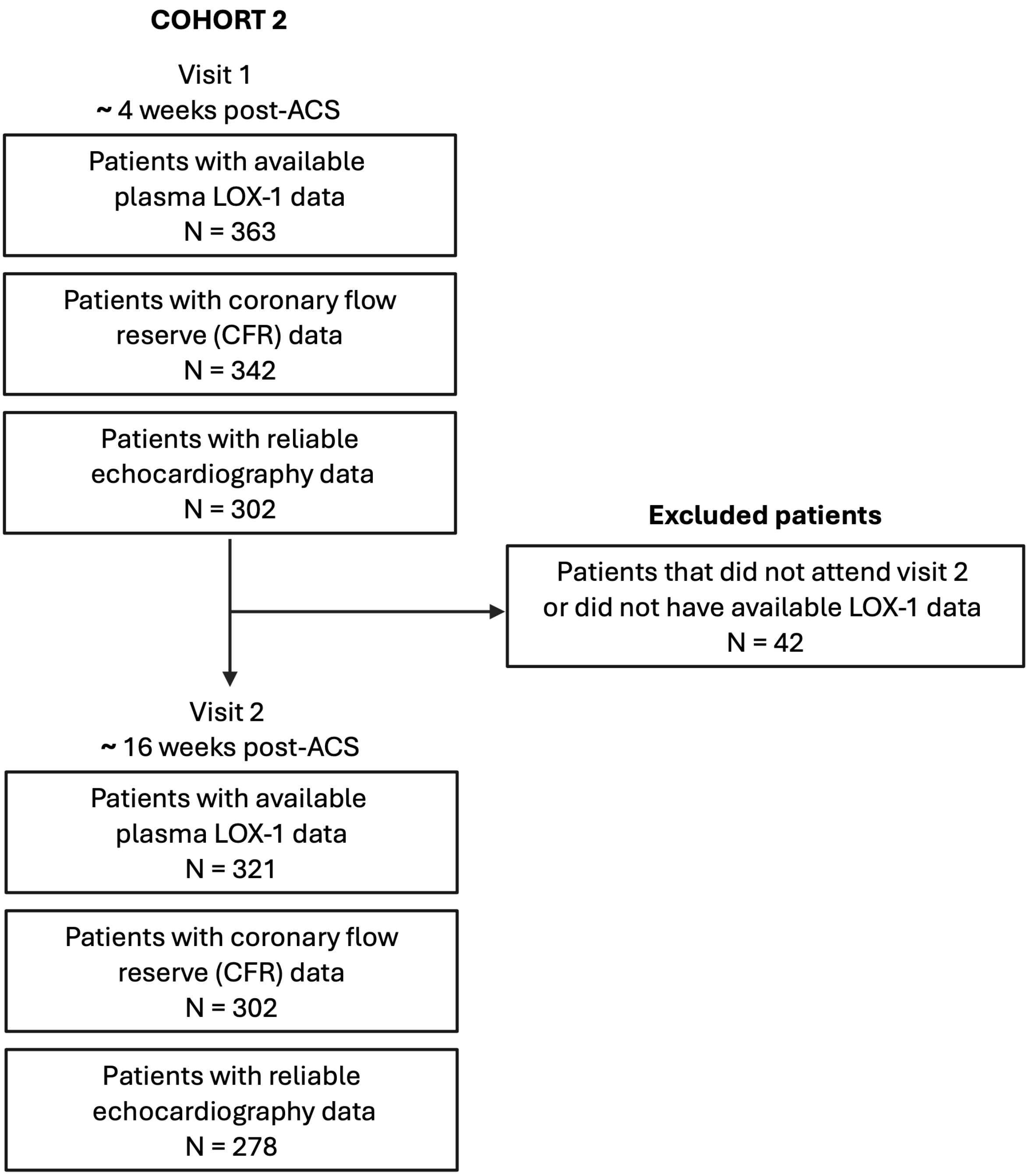

Supplement: Supplementary file 2 [file heartjnl-112-18-s004.pdf]
