# Supplementary material for: Circulating soluble LOX-1 and patient prognosis after an acute coronary syndrome
Source: Heart. 2025 Sep 16;112(18):e326315. doi: 10.1136/heartjnl-2025-326315 (PMC13539867; doi:10.1136/heartjnl-2025-326315)
Supplement: Supplementary file 3 [file heartjnl-112-18-s001.docx]

**Circulating soluble LOX-1 and Patient Prognosis After an Acute Coronary Syndrome**

Alexandru Schiopu, Sara Svedlund, Gayathri Narasimhan, Bi Juin Loong, Troels Yndigegn, Vijayalakshmi Varma, Emily L. Ongstad, Isabel Goncalves, Anna Collén, Jan Nilsson, Li-Ming Gan

**Supplemental material**

**Detailed methods**

***Study populations and outcomes***

The study included two different cohorts of ACS patients. The first cohort (cohort 1) has been previously described [1] and includes 524 consecutively enrolled patients with available baseline plasma samples collected within 24 hours of admission with a conformed ACS diagnosis (Supplemental figure 1). The patients have been recruited at the Coronary Care Unit of Skåne University Hospital Malmö, Sweden between October 2008 and December 2012. ACS was defined as unstable angina or acute MI, according to the universal definition [2]. Baseline clinical data were collected from the national Swedish Web-based system for Enhancement and Development of Evidence-based care in Heart disease Evaluated According to Recommended Therapies (SWEDEHEART). Cohort 1 was followed prospectively for a median [interquartile range (IQR)] period of 2.20 (1.15 – 3.27) years for incident HF and 2.08 (1.07 – 3.14) years for incident MACE. Participants were followed from baseline until the first occurrence of the outcome of interest, death, or the end of the follow-up period (December 31st, 2012), whichever came first. Participants who did not experience an event were censored at the time of death or at the end of follow-up. Time-to-event analyses were performed using Cox proportional hazards models, accounting for censoring. MACE was defined as a composite of hospitalization with an ACS diagnosis or cardiovascular (CV) death. Data from the Swedish Hospital Discharge Register and the Swedish Cause of Death Register were used to identify events. MI was defined by ICD 10 codes I21 and I22, unstable angina by code I20, and HF by code I50. CV death was defined as death due to MI, ischemic heart disease, cardiac arrest, atrial fibrillation, HF, and ischemic or hemorrhagic stroke, defined by the ICD10 codes I20-25, I46, I48, I50, and I60-69.

The second cohort (cohort 2) consisted of 363 ACS patients who had undergone acute percutaneous intervention (PCI) at index hospitalization at Sahlgrenska University Hospital, Gothenburg, Sweden between February 2010 and October 2013. The patients underwent follow-up examinations of cardiac function and coronary flow reserve by echocardiography, together with collection of plasma samples, within 4 and 16 weeks of PCI (Supplemental figure 2). Patients who were unable to comply with the study protocol were excluded.

All patients signed an informed consent form before inclusion in the studies. The first study (cohort 1) was approved by the Regional Ethics Committee for human studies in Lund, Sweden (reference number 211/2007). The second study (cohort 2) was approved by the Regional Ethics Committee for human studies in Gothenburg, Sweden (reference number 494-09). The studies were conducted according to the ethical guidelines of the Declaration of Helsinki.

***Patient and public involvement***

Patients have contributed with clinical data and blood samples and have undergone follow-up echocardiographic examinations for research purposes. Patients and/or the public were not involved in the design, reporting or dissemination of this research.

***Biochemical analysis***

Plasma samples were collected in EDTA-coated tubes, centrifuged at 3000 g for 10 minutes, aliquoted, and stored at –80°C. For cohort 1, high-sensitive troponin T (hsTnT) and cystatin C levels were analyzed at the clinical laboratory of Skåne University Hospital, Malmö, Sweden. The Chronic Kidney Disease Epidemiology Collaboration formula was used to calculate estimated glomerular filtration rate (eGFR) based on cystatin C, age, and sex [3].

For both study cohorts, the analysis of sLOX-1 in plasma was performed at the Science for Life Laboratory, Uppsala, Sweden, by the Proximity Extension Assay technique, as previously described [1, 4]. Data for sLOX-1 are presented as arbitrary units (au).

***Echocardiography***

To prospectively study cardiac recovery post-ACS, a subgroup of patients ≥75 years of age from cohort 1 underwent echocardiography imaging 1 year after inclusion. The pre-defined purpose of this sub-study was to assess post-ACS cardiac function recovery in the elderly. As previously described [1], the patients included in this detailed follow-up group had significantly lower body mass index (BMI), lower eGFR, lower prevalence of smoking, and higher prevalence of hypertension at baseline versus the rest of the cohort. The echocardiographic follow-up was completed in 113 patients (Supplemental figure 1). The echocardiography examinations were performed by experienced sonographers and were analyzed using the Xcelera software (Philips) by a single examiner blinded to the clinical data. The LV ejection fraction (LVEF), LV end-systolic volume (LVESV), and LV end-diastolic volume (LVEDV) were measured according to Simpson’s biplane method in the apical 4-chamber and 2-chamber views. The echocardiographic parameters were reliably measured in 74 patients at baseline and 97 patients at 1 year. Data from the other patients were excluded from the analysis due to poor image quality and missing frames (Supplemental figure 1).

In cohort 2, echocardiography examinations were performed at 4 and 16 weeks post-ACS to assess coronary flow reserve (CFR) in the left anterior descending artery (LAD), cardiac volumes and function (Supplemental figure 2). CFR assessment using color Doppler echocardiography is a measure of coronary microvascular function in absence of stenotic coronary artery lesions [5] and is a strong predictor of CV events in various populations [6]. The distal part of LAD was identified in a modified 2–3 chamber view using color Doppler. Baseline flow velocities were assessed using pulsed wave Doppler. Hyperemia was induced by continuous infusion of adenosine (140 µg/min/kg) for 5 minutes and peak velocity signals were detected. CFR was calculated as the ratio between maximal flow velocity at stress and flow velocity at rest. The protocol for CFR-examinations was described previously [5]. LVEF, LVESV, and LVEDV were measured according to Simpson’s biplane method in the apical 4-chamber and 2-chamber views. Out of the 363 patients with available sLOX-1 data at the 4 weeks visit, CFR could be measured in 342 patients and 302 patients had reliable echocardiography data for Simpson’s biplane method measurements. Out of the 321 patients with available sLOX-1 data at the 16 weeks visit, CFR was measured in 302 patients and reliable echocardiography data was available in 278 patients (Supplemental figure 2). All data were measured offline in SyngoDynamics (Siemens-Acuson, Mountain View, California).

***Cell culture***

Human umbilical vein endothelial cells (HUVECs) were grown in Medium 200 containing 2% low serum growth supplement (Invitrogen) with addition of 1% penicillin-streptomycin (Gibco) until confluency (90-95%) in 24-well plates. Before stimulation, the cells were starved with 0.5% serum in Medium 200 for 24 hours. HUVECs were stimulated with oxLDL (30 μg/mL) for 24 hours in a 37°C incubator with 5 % CO_2_ in starvation media. oxLDL was prepared by exposure to 5 μmol/L CuSO4 for 18 hours at 37°C as previously described [7]. Cellular LOX-1 activation was blocked by addition of a polyclonal IgG antibody against the C-terminal end of the protein, which is responsible for oxLDL recognition and binding (5 μg/mL and 10 μg /mL, Abcam, ab203246).

***Quantitative real-time PCR (qRT-PCR)***

After 24 hours of treatment, cells were washed with ice-cold PBS and homogenized in RLT buffer (Qiagen). Total RNA was extracted using a RNeasy mini kit (Qiagen). cDNA was synthesized using a high-capacity RNA-to-cDNA kit (Applied Biosystems). mRNA expression of target genes was determined using the Taqman Universal PCR master mix (Applied Biosystems) and Taqman gene expression assays for fibronectin 1 (*Fn1*, Hs01549976_m1), ICAM1 (*Icam1,* Hs00164932_m1), P-selectin (*Selp*, Hs00927900_m1), L-selectin (*Sell,* Hs00174151_m1) and E-selectin (*Sele*, Hs00174057_m1) (Applied Biosystems) on a QuantStudio^TM^ 7 Flex system. Gene expression was determined by the 2^-ddCT comparative analysis method and represented as fold change normalized to no-oxLDL controls. GAPDH was used as a housekeeping gene (Applied Biosystems).

***Statistical analysis***

Data distribution has been assessed by the Kolmogorov-Smirnov test. As some of the variables were not normally distributed, we used non-parametric tests to assess differences between groups and correlations between variables. Non-normally distributed variables were log-transformed and standardized as Z-scores before being entered in the multivariable linear regression and the Cox regression analyses. The Mann–Whitney test for continuous variables and the chi-squared test for dichotomous variables were used to compare clinical and biochemical characteristics between the outcome groups. The correlations between baseline sLOX-1, hsTnT, and CV risk factors at baseline were examined by the Spearman’s rank test and by multivariable linear regression analyses. Kaplan–Meier curves and multivariable Cox proportional hazards analyses were used to assess the associations between baseline sLOX-1 and outcomes. For the Kaplan-Meier analysis, the subjects were divided into tertiles based on baseline plasma sLOX-1 values. We used three different adjustment models for the Cox proportional hazards analyses: Model 1 was adjusted for age and sex; Model 2 included age, sex, hypertension, diabetes mellitus, smoking, eGFR, previous HF, and/or ischemic heart disease; Model 3 additionally accounted for differences in revascularization and treatment at discharge. The Cox proportional hazards models were adjusted for these potential confounders as they differed significantly between outcome groups at baseline (Table 1). Although sex and smoking status did not differ between groups, they were included in the models due to their established impact on post-ACS prognosis. Additionally, smoking was independently associated with plasma sLOX-1 levels in cohort 1 (Supplemental Table 1). The correlations between sLOX-1, biomarkers and echocardiographic parameters in cohort 2 were analyzed with the Spearman’s rank test. Patients with missing variables were excluded from the respective analysis. All hypothesis testing was conducted using two-tailed significance tests. Two-tailed *P* values <0.05 were considered significant. The calculations were performed using SPSS 23.0 (IBM software, Armonk, New York). One-way ANOVA analysis was performed using GraphPad Prism 10 for the gene expression studies.

**References**

1	Grauen Larsen H, Yndigegn T, Marinkovic G, et al. The soluble receptor for advanced glycation end-products (sRAGE) has a dual phase-dependent association with residual cardiovascular risk after an acute coronary event. *Atherosclerosis* 2019;**287**:16–23.

2	Thygesen K, Alpert JS, Jaffe AS, et al. Third universal definition of myocardial infarction. *Glob Heart* 2012;**7**:275–95.

3	Zamora E, Lupón J, de Antonio M, et al. Long-term prognostic value for patients with chronic heart failure of estimated glomerular filtration rate calculated with the new CKD-EPI equations containing cystatin C. *Clin Chem* 2014;**60**:481–9.

4	Assarsson E, Lundberg M, Holmquist G, et al. Homogenous 96-plex PEA immunoassay exhibiting high sensitivity, specificity, and excellent scalability. *PLoS One* 2014;**9**:e95192.

5	Shah SJ, Lam CSP, Svedlund S, et al. Prevalence and correlates of coronary microvascular dysfunction in heart failure with preserved ejection fraction: PROMIS-HFpEF. *Eur Heart J* 2018;**39**:3439–50.

6	Gan LM, Svedlund S, Wittfeldt A, et al. Incremental Value of Transthoracic Doppler Echocardiography-Assessed Coronary Flow Reserve in Patients With Suspected Myocardial Ischemia Undergoing Myocardial Perfusion Scintigraphy. *J Am Heart Assoc* 2017;**6**.

7	Niemann-Jonsson A, Dimayuga P, Jovinge S, et al. Accumulation of LDL in rat arteries is associated with activation of tumor necrosis factor-alpha expression. *Arterioscler Thromb Vasc Biol* 2000;**20**:2205–11.
