# Supplementary material for: Circulating soluble LOX-1 and patient prognosis after an acute coronary syndrome
Source: Heart. 2025 Sep 16;112(18):e326315. doi: 10.1136/heartjnl-2025-326315 (PMC13539867; doi:10.1136/heartjnl-2025-326315)
Supplement: Supplementary file 4 [file heartjnl-112-18-s005.docx]

**Circulating soluble LOX-1 and Patient Prognosis After an Acute Coronary Syndrome**

Alexandru Schiopu, Sara Svedlund, Gayathri Narasimhan, Bi Juin Loong, Troels Yndigegn, Vijayalakshmi Varma, Emily L. Ongstad, Isabel Goncalves, Anna Collén, Jan Nilsson, Li-Ming Gan

**Supplemental tables**

**Supplemental table 1.** Mutually adjusted relationships between plasma sLOX-1 concentration and CV risk factors at baseline in cohort 1

| **Risk factors** | **Beta coefficient** | **95% CI for the beta coefficient** | ***P*** | |
| --- | --- | --- | --- | --- |
| Age (per year) | 0.005 | –0.002, 0.011 | 0.150 |  |
| Male gender | –0.003 | –0.126, 0.120 | 0.967 |  |
| Hypertension | –0.021 | –0.135, 0.094 | 0.725 |  |
| Current smoking | 0.199 | 0.068, 0.330 | 0.003 |  |
| Diabetes | 0.031 | –0.101, 0.163 | 0.644 |  |
| ln(BMI) | 0.051 | –0.005, 0.108 | 0.076 |  |
| ln(eGFR) | –0.021 | –0.092, 0.051 | 0.572 |  |
| ln(hsTnT) | 0.022 | –0.034, 0.077 | 0.446 |  |
| Previous cardiac event |  |  |  |  |
| HF | 0.007 | –0.194, 0.207 | 0.948 |  |
| ACS | 0.061 | –0.074, 0.196 | 0.377 |  |

Multivariable linear regression analysis with sLOX-1 as a dependent variable and CV risk factors, eGFR, and hsTnT as independent variables. The beta coefficient for the continuous variables is expressed per 1-SD (standard deviation) increase in the respective biomarker. The values for BMI, eGFR, and hsTnT were log-transformed and standardized as Z-scores prior to analysis, to enable direct comparison of the beta coefficients across variables.

ACS, acute coronary syndrome; BMI, body mass index; CI, confidence interval; CV, cardiovascular; eGFR, estimated glomerular filtration rate; HF, heart failure; hsTnT, high-sensitive cardiac troponin T; sLOX-1, soluble lectin-like oxidized low-density lipoprotein receptor-1.

**Supplemental table 2.** Demographic data for cohort 2

| **Characteristics** | **All patients** |
| --- | --- |
|  | N=363 |
| Age, years, median (IQR) | 63 (57, 70) |
| Male gender, n (%) | 299 (82.4) |
| Hypertension, n (%) | 154 (42.4) |
| Smoking, n (%) | 132 (36.4) |
| Diabetes, n (%) | 43 (11.8) |
| BMI, kg/m^2^, median (IQR) | 27 (25, 29) |
| Previous MI, n (%) | 46 (12.7) |
| Coronary angiography, n (%) |  |
| Left main stenosis | 3 (<1) |
| 1-vessel no left main | 199 (54.8) |
| 2-vessel no left main | 114 (31.4) |
| 3-vessel no left main | 35 (9.6) |
| Non-significant stenosis | 0 |
| Revascularization, n (%) | 351 (99.7) |
| PCI | 301 (82.9) |
| CABG | 50 (13.8) |
| TnT (ng/L), median (IQR) | 18 (10, 63) |
| sLOX-1, au, median (IQR) | 5.6 (5.2, 5.9) |

IQR, interquartile range; ACS, acute coronary syndrome; au, arbitrary units; BMI, body mass index; CABG, coronary artery bypass graft; MI, myocardial infarction; PCI, percutaneous coronary intervention; sLOX-1, soluble lectin-like oxidized low-density lipoprotein receptor-1; TnT, troponin T; SD, standard deviation.

**Supplemental table 3.** Mutually adjusted relationships between plasma sLOX-1 concentration and CV risk factors at the 4 weeks follow-up visit in cohort 2

| **Risk factors** | **Beta coefficient** | **95% CI for the beta coefficient** | ***P*** |
| --- | --- | --- | --- |
| Age (per year) | 0.008 | –0.001, 0.016 | 0.082 |
| Male gender | –0.246 | –0.437, 0.054 | 0.012 |
| Hypertension | –0.005 | –0.158, 0.148 | 0.949 |
| Current smoking | 0.020 | –0.153, 0.193 | 0.823 |
| Diabetes | –0.105 | –0.329, 0.118 | 0.353 |
| BMI (kg/m^2^) | 0.167 | –0.390, 0.725 | 0.554 |
| eGFR (mL/min/1.73m^2^) | 0.193 | –0.146, 0.533 | 0.263 |
| hsTnT (ng/L) | 0.019 | –0.031, 0.068 | 0.456 |
| Previous cardiac event |  |  |  |
| HF | 0.079 | –0.238, 0.395 | 0.624 |
| ACS | –0.046 | –0.355, 0.264 | 0.771 |

Multivariable linear regression analysis with plasma sLOX-1 as dependent variable and CV risk factors as independent variables.

ACS, acute coronary syndrome; BMI, body mass index; CI, confidence interval; CV, cardiovascular; eGFR, estimated glomerular filtration rate; HF, heart failure; hsTnT, high-sensitive troponin T; sLOX-1, soluble lectin-like oxidized low-density lipoprotein receptor-1.
